# Physiology

**Published:** 1878-11

**Authors:** 


*Collaborators:*

Dr. H. Gradle, Dr. L. W. Case, Dr. R. Park,
Dr. R. Tilley, Dr. D. R. Brower.

### Physiology.

The Physiology of Labyrinthine Vertigo. (*The Doctor*, Sept. 1, 1878.)

Some recent papers by Dr. Woakes have been thus summarized by Dr. Orne Green, for the *Boston Med. Jour.* The tympanum is supplied with arterial blood from the carotid, and its veins discharge into the jugular. The labyrinth is supplied by the vertebral, and its veins discharge into the superior petrosal sinus. The vertebral artery derives many of its vaso-motor nerves from the inferior cervical ganglion, and these nerves communicate with the brachial plexus. The same ganglion furnishes the inferior cardiac nerve, the principal inhibitory nerve of the heart. Besides these connections a fasciculus is given off from the pneumogastric, near the recurrent laryngeal, to the lower cervical ganglion. So that we have in this ganglion an organ connecting the upper extremities, heart and upper portion of the digestive tract with the labyrinthine circulation. From any irritation we may have a diminished inhibition which shall relax the walls of the vertebral artery and cause pressure on the endolymph, i. e. vertigo. Hence such vertigo does not necessarily imply previous disease of the ear.

Weir Mitchell has noticed that gunshot wounds of the upper extremities are often followed by dizziness.

Quinine in large doses and tobacco diminish vascular tonus; while bromine, the bromides and especially hydrobromic acid stimulate vascular innervation. Hence the antagonistic effect of the latter on the tinnitus caused by the former.

